# Fish Allergy Around the World—Precise Diagnosis to Facilitate Patient Management

**DOI:** 10.3389/falgy.2021.732178

**Published:** 2021-10-13

**Authors:** Tanja Kalic, Christian Radauer, Andreas L. Lopata, Heimo Breiteneder, Christine Hafner

**Affiliations:** ^1^Department of Dermatology, University Hospital St. Poelten, Karl Landsteiner University of Health Sciences, St. Poelten, Austria; ^2^Center for Pathophysiology, Infectiology and Immunology, Institute of Pathophysiology and Allergy Research, Medical University of Vienna, Vienna, Austria; ^3^Molecular Allergy Research Laboratory, Australian Institute of Tropical Health and Medicine, James Cook University, Townsville, QLD, Australia; ^4^Tropical Futures Institute, James Cook University, Singapore, Singapore; ^5^Karl Landsteiner Institute for Dermatological Research, Karl Landsteiner Society, St. Poelten, Austria

**Keywords:** fish allergy, fish allergen diversity, fish extracts, molecular allergy diagnosis, patient management, cross-reactivity

## Abstract

The accurate and precise diagnosis of IgE-mediated fish allergy is one of the biggest challenges in allergy diagnostics. A wide range of fish species that belong to evolutionary distant classes are consumed globally. Moreover, each fish species may contain multiple isoforms of a given allergen that often differ in their allergenicity. Recent studies indicated that the cross-reactivity between different fish species is limited in some cases and depends on the evolutionary conservation of the involved allergens. Fish allergens belong to several protein families with different levels of stability to food processing. Additionally, different preparation methods may contribute to specific sensitization patterns to specific fish species and allergens in different geographic regions. Here, we review the challenges and opportunities for improved diagnostic approaches to fish allergy. Current diagnostic shortcomings include the absence of important region-specific fish species in commercial *in vitro* and *in vivo* tests as well as the lack of their standardization as has been recently demonstrated for skin prick test solutions. These diagnostic shortcomings may compromise patients' safety by missing some of the relevant species and yielding false negative test results. In contrast, the avoidance of all fish as a common management approach is usually not necessary as many patients may be only sensitized to specific species and allergens. Although food challenges remain the gold standard, other diagnostic approaches are investigated such as the basophil activation test. In the context of molecular allergy diagnosis, we discuss the usefulness of single allergens and raw and heated fish extracts. Recent developments such as allergen microarrays offer the possibility to simultaneously quantify serum IgE specific to multiple allergens and allergen sources. Such multiplex platforms may be used in the future to design diagnostic allergen panels covering evolutionary distant fish species and allergens relevant for particular geographic regions.

## Introduction

Based on a position paper published by the European Academy of Allergy and Clinical Immunology (EAACI), allergic hypersensitivity reactions, including those to foods, cover IgE- and non-IgE-mediated adverse reactions of the immune system (1). The most common type of hypersensitivity reactions to fish is IgE-mediated fish allergy. Allergic reactions to fish mediated by allergen-specific IgE occur immediately after the exposure and are characterized by a variety of symptoms ranging from mild, including local urticaria and oral allergy syndrome, to serious such as severe anaphylaxis. Some of the well-known non-IgE mediated food hypersensitivities include food protein- induced allergic proctocolitis (FPIAP) and food protein-induced enterocolitis syndrome, and eosinophilic esophagitis (2). Fish is one of the main causative agents of FPIAP. Symptoms of non-IgE mediated hypersensitivity occur hours or days after the exposure to fish. These reactions are difficult to diagnose due to the lack of specific tests (2, 3). Although these adverse reactions are etiologically different from IgE-mediated fish allergy, they might be confused with one another and should be considered during the diagnostic workup of the patients reporting allergy-like reactions upon consumption of fish.

Diagnosis of IgE-mediated fish allergy is usually done by assessing the clinical history, performing skin testing using commercially available fish extracts, and quantifying IgE specific to fish extracts or the major allergen parvalbumin. In addition, prick-to-prick tests using fresh or cooked fish are sometimes used and they overcome the problem of lack of available skin test extracts, which is an increasing regulatory and quality problem especially within the EU. The gold standard and the only way to confirm or exclude allergy to specific species with certainty is the double-blind placebo-controlled food challenge (DBPCFC) (3). The diagnosis of fish allergy is more complex than of many other allergies due to the variety of fish species, the amount of allergens they contain, the allergens' degree of evolutionary distance to human homologs, and the fish processing methods which may alter allergen composition and IgE-reactivity (4). Fish consumed globally belong to hundreds of species from different fish families. In addition, different consumption habits in different parts of the world, associated with the diverse fish processing methods, may alter the local sensitization patterns. Considering the variety of fish species and allergens, a one-fits-all approach for fish allergy diagnosis is not suitable for the patients. Previous research indicated that up to 29% of physician-diagnosed fish allergy patients may be able to safely consume some of the fish species that they are not sensitized to (5). In this study, 35 subjects underwent DBPCFC with cod, salmon, and mackerel, and tolerance of at least one of the species was detected in 10 participants. However, current clinical practice usually advises patients to generally avoid all fish.

This review focuses on the complexity of IgE-mediated fish allergy and discusses different aspects that need to be considered for the improvement of diagnosis and patient management.

## Diversity of Consumed Fish and Its Impact on Allergy Diagnosis

### Fish Consumption and Allergy Prevalence

Consumption of fish is steadily on the increase worldwide due to their high nutritional value. While the extent of global fishing seems to remain constant, fish farming in aquaculture is rapidly growing (6). It has recently been suggested that countries with a high consumption of fish also have a higher prevalence of fish allergy in comparison to regions where fish consumption is less common. Most of the recently published epidemiological studies on fish allergy focused on pediatric allergic patients (summarized in Table 1). However, determining the prevalence of true fish allergy can be difficult as in many cases immunological data are unavailable. Often the prevalence of fish allergy is estimated by discussing the clinical history with the patients or by evaluating questionnaires (21). Such studies carry the risk of overestimating the incidence of IgE-mediated fish allergy as many allergy-like reactions may occur due to other factors such as scombroid poisoning, exposure to fish toxins, to fish parasite Anisakis or adverse reactions to fish mediated by non-IgE related mechanisms such as FPIAP (22).

**Table 1:** Fish allergy prevalence and fish consumption.

**Country**	**Adult (A)/children (C)**	**Questionnaire-based prevalence (%)**	**Clinically confirmed fish allergy prevalence (%)**	**Reference**	Consumption (kg/capita/year)^*^
**North America**					
United States	C	0.3–0.6	–	Gupta et al. (7)	12.3
United States	A	0.9	–	Gupta et al. (8)	12.3
**Asia**					
China	C	–	0.2	Chen et al. (9)	21.1
Israel	C	0.01	–	Dalal et al. (10)	22.2
Philippines	C	2.3	–	Connet et al. (11)	28.5
UAE	C	2.8	–	Al-Hammadi et al. (12)	20.4
Singapore	C	0.3	–	Connet et al. (11)	–
Thailand	C	0.3	–	Connet et al. (11)	19.4
Vietnam	C	1.6	1.2	Le Thu et al. (13)	26.5
**Africa**					
Ghana	C	0.3	–	Obeng et al. (14)	26.0
**Europe**					
Finland	C	5–7	0.3	Pyrhonen et al. (15)	33.8
France	C	–	0.7	Penard-Morand et al. (16)	22.9
Germany	C	0.2	0.6	Grabenhenrich et al. (17) and Schnabel et al. (18)	11.2
Iceland	C	1.3	–	Grabenhenrich et al. (17)	74.4
Lithuania	C	0.5	–	Grabenhenrich et al. (17)	42.4
Netherlands	C	0.3	–	Grabenhenrich et al. (17)	18.6
Norway	C	3	–	Eggesbo et al. (19)	38.2
Poland	C	0.4	–	Grabenhenrich et al. (17)	10.5
Sweden	C	–	0.7	Ostblom et al. (20)	23.9
UK	C	0.3	–	Grabenhenrich et al. (17)	15.5

* *Consumption is based on FAOSTAT database on Food Supply - Livestock and Fish Primary Equivalent for 2013 (www.fao.org/faostat/en/#data/CL, accessed on June 20th 2021)*. *–, not available; C, children; A, adults*.

Based on the currently available literature, the prevalence of fish allergy according to clinical history and questionnaires is the lowest in Israel (0.01%) (10), followed by several countries such as Singapore, Thailand and Ghana with prevalence between 0.2 and 0.3% (11, 14) (Table 1). In the US, Gupta et al. determined a prevalence of fish allergy of 0.3–0.6% for children depending on the age, and up to 0.9% for adults (7, 8). Among the Asian countries, the highest prevalence of reported fish allergy was found in the Philippines (2.3%) and Vietnam (1.6%) (11, 13, 23). In the EU, Finland is the country with the most commonly reported fish allergy of up to 7%, followed by Norway (3%) (15, 19). Recently published results from the EuroPrevall-iFAAM birth cohort, which reported the frequency of food allergy in school-aged children in eight European countries, showed average sensitization rates to any fish between 0.2% in Germany and 1.3% in Iceland (Table 1) (17).

However, when looking into clinically confirmed fish allergy, the rates are usually lower (Table 1). To get an insight into the correlation between fish consumption rates and reported allergy, we extracted data on fish consumption (per capita per year) from the reports of the Food and Agriculture Organization of the United Nations (FAO) for all the countries for which we found available data on fish allergy prevalence (Table 1). Based on the presented studies, we could not observe a correlation between the consumption rates and the prevalence of clinically confirmed IgE sensitization to fish (Spearman's *R* = 0.29, *P* = 0.6; calculated in GraphPad Prism 9.1). Of note, the lack of correlation observed for specific cohorts from large countries such as US and China should not be extrapolated to the whole countries, as differences may be observed between inland and coastal regions. Hence, a larger number of studies on the fish allergy incidences in different countries are required to make a more certain conclusion.

### Diversity of Consumed Fish

Fish consumed around the world belong to a wide range of species often from evolutionary distant classes. Well-known allergens from different fish species frequently share between 50 and 75% of sequence identities resulting in cross-reactivity to different species for many fish-sensitized individuals (24).

**Figure 1:**
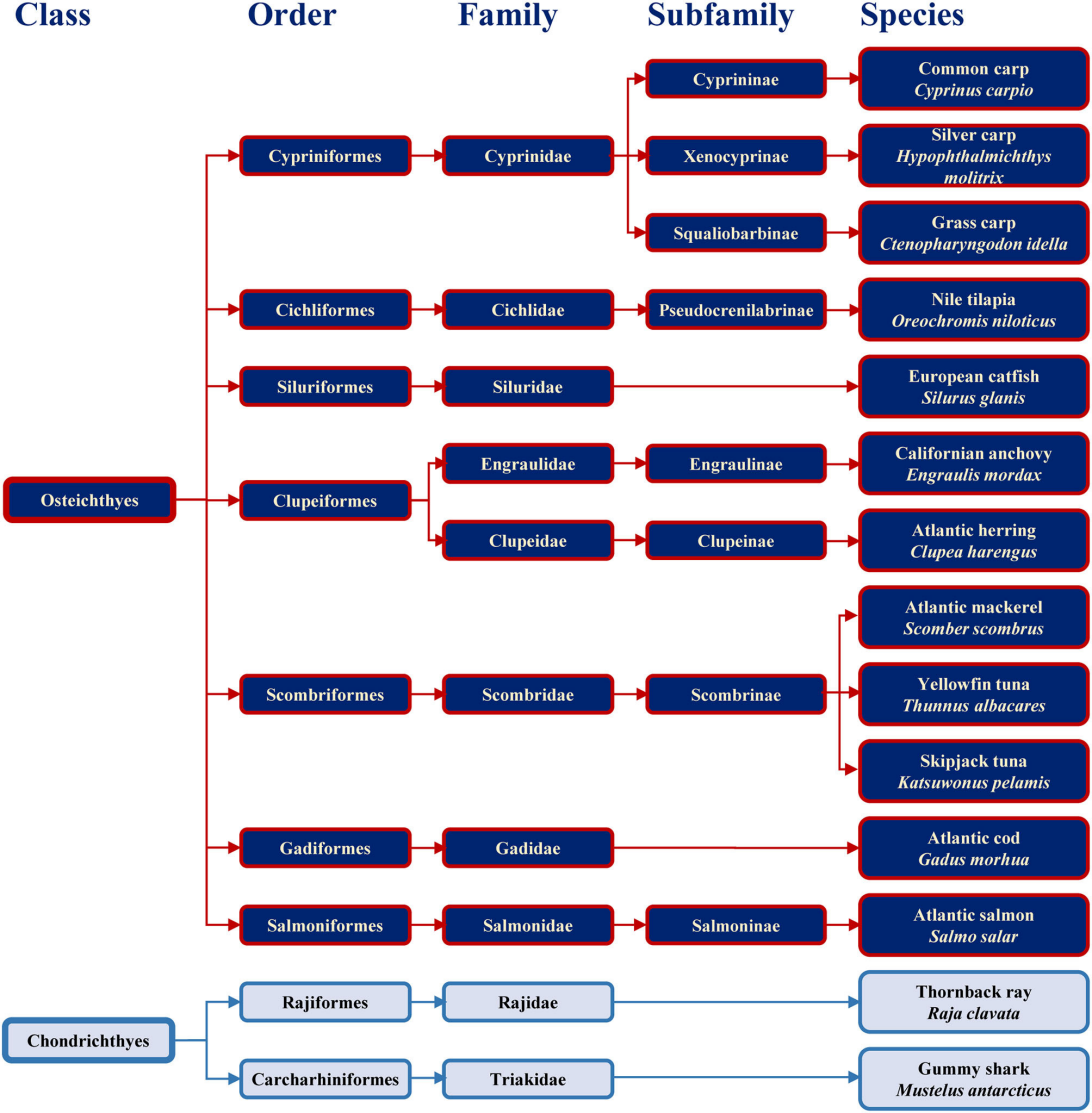
Classification of the main bony and cartilaginous fish species relevant for human consumption.

Families containing some of the most commonly consumed fish are shown in Figure 1 together with example species. The consumed species belong to bony fish (Osteichthyes) or to cartilaginous fish (Chondrichthyes). Cartilaginous fish are a class comprising fish with skeletons composed of cartilage and are divided into the two subclasses of Holocephali (ghost sharks) and Elasmobranchii (rays, sharks, skates and sawfish). All frequently consumed cartilaginous fish shown in Figure 1 belong to Elasmobranchii. The wider variety of commonly consumed species belong to bony fish, which are a diverse taxonomic group containing ray-finned fish (Actinopterygii) and lobe-finned fish (Sarcopterygii) (25). All bony fish species presented in Figure 1 belong to Actinopterygii.

Previous study showed the absence of IgE-binding and basophil activation upon exposure to parvalbumin from cartilaginous fish, and the tolerance of cartilaginous fish upon its consumption by majority of patients (10 of 11) sensitized to bony fish (26). Fish extracts and purified allergens from evolutionary distant species should be considered when setting up diagnostic approaches with the aim to safely conclude which species a patient may react to and which may be tolerated.

### Fish Production and Trade

The trade in fish and fish products is global and makes non-native species available for consumption in different geographic regions. In a recent publication, the FAO expects the world fish trade to grow by 9% until 2030, reaching over 54 million tons in live weight annually (27). Currently, as reported by the European Market Observatory for Fisheries and Aquaculture (www.eumofa.eu), the EU and the US are the fifth and the sixth largest fish producers worldwide, respectively, following China, Indonesia, India and Vietnam. In the EU, ~20% of the fish come from aquaculture while the rest is from fisheries. The main fish producers in Europe are Spain, Denmark, the United Kingdom, and France (www.eumofa.eu, accessed on July 28th 2021).

**Figure 2:**
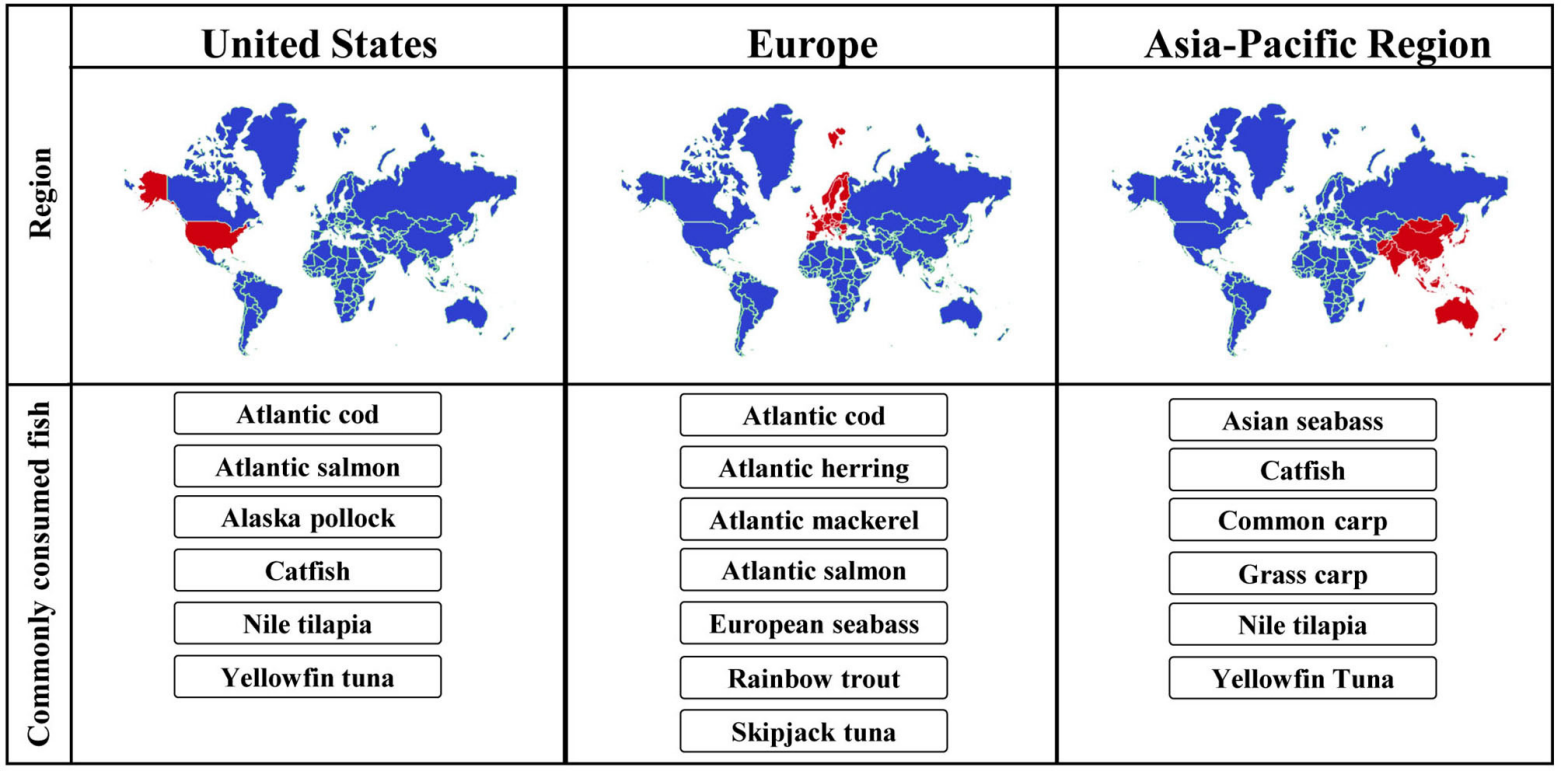
Most commonly consumed fish species in different geographic regions.

Although the fish trade worldwide is high, the most commonly produced and consumed species differ in different geographic regions. Some of the most relevant species for specific regions are depicted in Figure 2. In many Asia-Pacific countries, Nile tilapia, several carp species, catfish and tuna are consumed predominantly (28, 29). Based on the reports by the European commission, Atlantic herring, Atlantic mackerel, sardines, Atlantic cod and skipjack tuna were among the top ten fish species caught in the EU in 2017, while the most common species raised in aquaculture were Atlantic salmon, rainbow trout and the European seabass (27). In the US, the most commonly consumed species are farmed Atlantic salmon, catfish and tilapia (30).

For an accurate diagnosis of fish allergy, the species most relevant for particular geographic regions should be considered. For example, while several species of carp are frequently consumed in some countries of Asia, they do not belong to the most frequently consumed fish in Europe and the US. Due to a limited cross-reactivity to phylogenetically distant species (31), diagnostic tests that focus on species not relevant for the local patient population may be misleading.

## The Variety of Fish Allergens as a Challenge for Diagnosis

The first discovered fish allergens were Gad m 1 and Gad c 1 (previously called Allergen M), β-parvalbumins from Atlantic and Baltic cod, respectively (32, 33). Since then, homologous allergens were identified as major allergens in multiple fish species. To date (June 2021), the WHO/IUIS Allergen Nomenclature Sub-Committee (www.allergen.org) has recognized β-parvalbumins from 16 fish species as allergens (Table 2). In recent years, however, members of several other protein families were identified as minor fish allergens (Table 2). These additional allergens may play a role in refining the diagnosis for certain patients.

**Table 2:** Families of fish allergens.

**Protein family**	**Molecular mass (kDa)**	**Frequency of IgE binding (%)**	**Number of registered allergens**	**Example**
β-Parvalbumin	12	80–100	16	Gad m 1 (*Gadus morhua*, Atlantic cod)
β-Enolase	47	20–60	5	Sal s 2 (*Salmo salar*, Atlantic salmon)
Aldolase A	40	10–40	4	Thu a 3 (*Thunnus albacares*; yellowfin tuna)
Tropomyosin	33	6–34	3	Ore m 4 (*Oreochromis mossambicus*; Mozambique tilapia)
Collagen type I α1-chain	130–140	22	2	Lat c 6 (*Lates calcarifer*; Barramundi)
Creatine kinase	43	10–14	2	Sal s 7 (*Salmo salar*, Atlantic salmon)
Triosephosphate isomerase	27	20–30	2	Pan h 8 (*Pangasianodon hypophthalmus*; striped catfish)
Pyruvate kinase	59	8	1	Pan h 9 (*Pangasianodon hypophthalmus*; striped catfish)
Lactate dehydrogenase	36	15	1	Pan h 10 (*Pangasianodon hypophthalmus*; striped catfish)
Glucose 6-phosphate isomerase	62	6	1	Pan h 11 (*Pangasianodon hypophthalmus*; striped catfish)
Glyceraldehyde-3-phosphate dehydrogenase	37	8	1	Pan h 13 (*Pangasianodon hypophthalmus*; striped catfish)
Vitellogenin	19	100^a^	1	Onc k 5 (*Oncorhynchus keta*; chum salmon)

a *Among patients allergic to chum salmon roe*. *Data were compiled from the WHO/IUIS Allergen Nomenclature Database (www.allergen.org, accessed on June 25th 2021)*.

### Allergen Families

#### Parvalbumin

**Figure 3:**
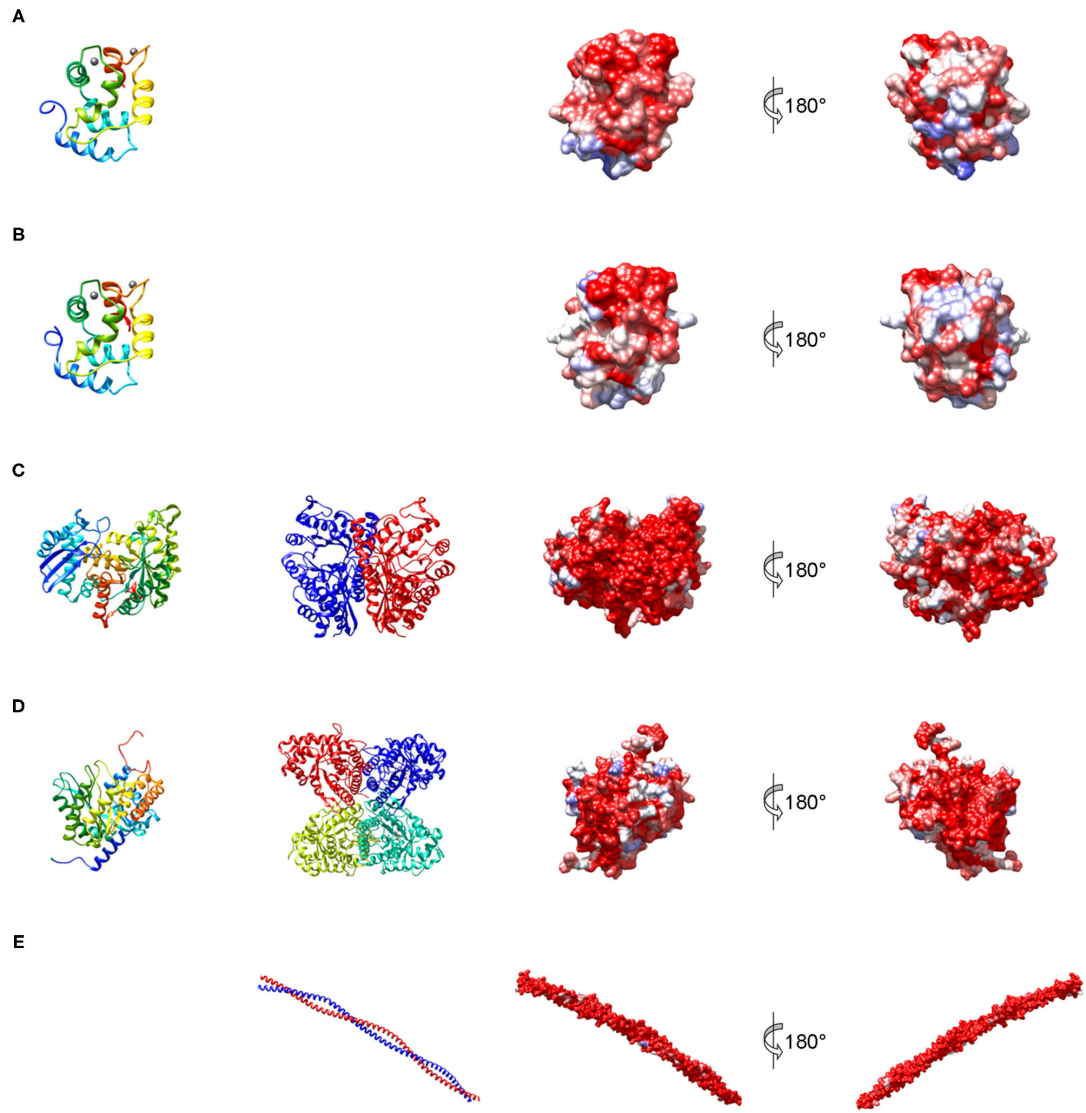
Structures representing the most important families of fish allergens. **(A)** β-Parvalbumin from common carp (PDB: 4cpv); **(B)** α-parvalbumin from Northern pike (PDB: 1pva); **(C)** human β-enolase (PDB: 2xsx); **(D)** aldolase A from rabbit (PDB: 1ado); **(E)** human tropomyosin α1 (PDB: 6x5z). Left column: secondary structure of a single subunit colored from blue (N-terminus) to red (C-terminus); middle column: quaternary structure; right columns: molecular surface colored by sequence conservation derived from a sequence alignment of vertebrate homologs from blue (different residues in all sequences) to red (identical residue in all sequences).

Parvalbumin, like calmodulin and troponin C, is a member of the EF hand-containing calcium binding protein superfamily. Parvalbumin is an acidic intracellular protein of low molecular weight and a key factor in regulating calcium homeostasis in fast-twitch muscle fibers. The first calciprotein structure was described for carp parvalbumin in 1972 (34). The protein contains three calcium-binding motifs (AB, CD, and EF), each comprising a 12-residue loop inserted between two 8 to 9 residue long α helices (Figures 3A,B). In parvalbumin, only motifs CD and EF can bind calcium, as well as magnesium ions. Parvalbumin transports calcium from troponin-C to the sarcoplasmic reticulum calcium pump during muscle relaxation but also plays a role in neuronal activity (35). Parvalbumins are present in all vertebrates and are divided into three evolutionary sublineages, α-parvalbumins, β-parvalbumins and oncomodulins, which had originally been classified as mammalian β-parvalbumins (36).

**Figure 4:**
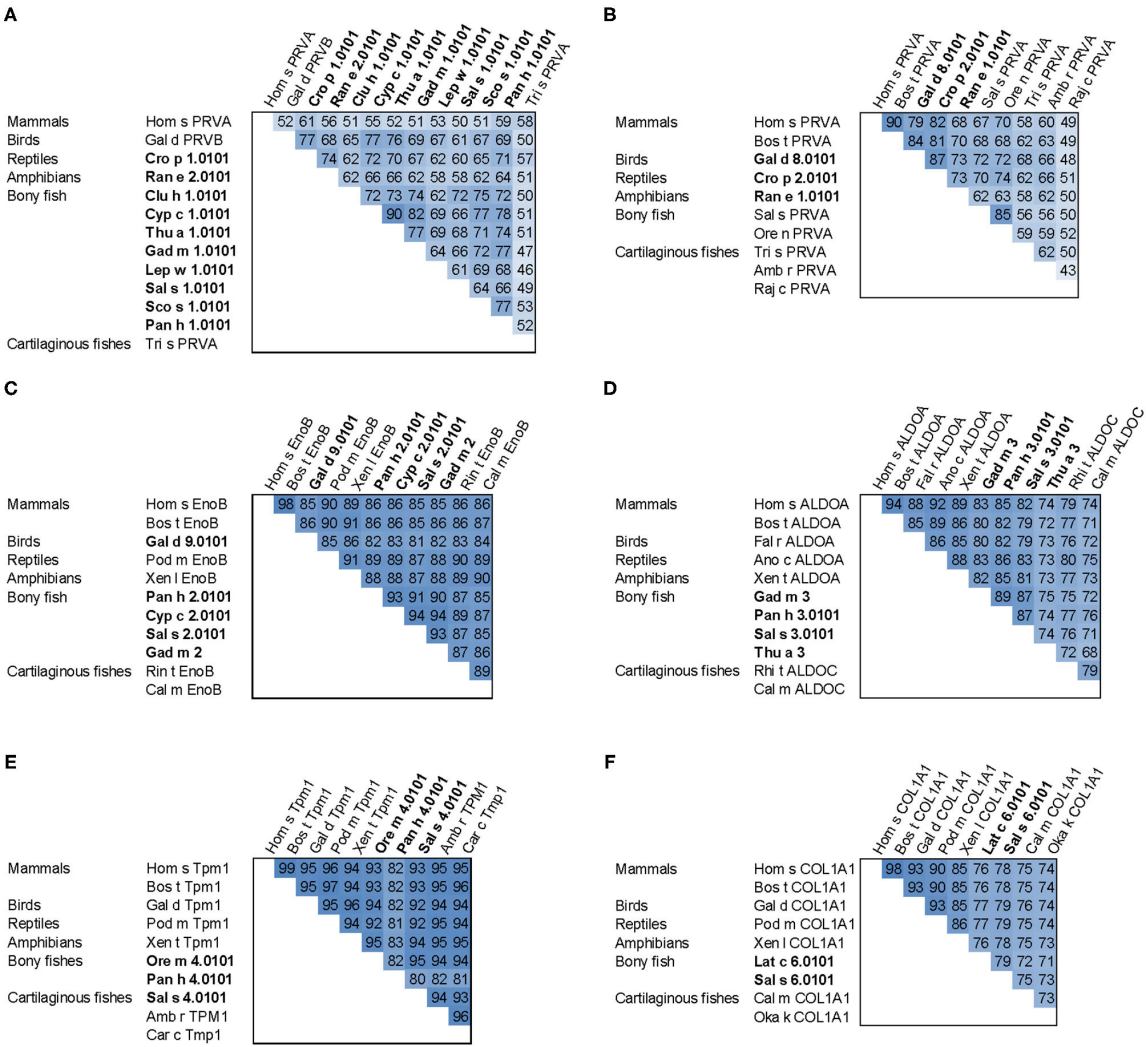
Sequence comparison of fish allergens with homologs from other vertebrates. **(A)** β-parvalbumins compared with human and cartilaginous fish α-parvalbumins; **(B)** α-parvalbumins; **(C)** β-enolase; **(D)** aldolase A compared with cartilaginous fish aldolase C; **(E)** tropomyosin α1; **(F)** collagen type I α1 chain. Allergens are printed in bold; the numbers show percent sequence identities between pairs of proteins extracted from multiple sequence alignments generated using ClustalX (37). Species abbreviations: Amb r, *Amblyraja radiata* (thorny skate); Ano c, *Anolis carolinensis* (American chameleon); Bos t, *Bos taurus* (cattle); Cal m, *Callorhinchus milii* (elephant shark); Car c, *Carcharodon carcharias* (great white shark); Clu h, *Clupea harengus* (Atlantic herring); Cro p, *Crocodylus porosus* (Australian saltwater crocodile); Cyp c, *Cyprinus carpio* (Common carp); Fal r, *Falco rusticolus* (gyrfalcon); Gad m, *Gadus morhua* (Atlantic cod); Gal d, *Gallus domesticus* (chicken); Hom s, *Homo sapiens* (human); Lat c, *Lates calcarifer* (barramundi perch); Lep w, *Lepidorhombus whiffiagonis* (megrim); Oka k, *Okamejei kenojei* (ocellate spot skate); Ore m, *Oreochromis mossambicus* (Mozambique tilapia); Ore n, *Oreochromis niloticus* (Nile tilapia); Pan h, *Pangasianodon hypophthalmus* (striped catfish); Pod m, *Podarcis muralis* (common wall lizard); Raj c, *Raja clavata* (thornback ray); Ran e, *Pelophylax lessonae* (Pool frog); Rhi t, *Rhincodon typus* (whale shark); Sal s, *Salmo salar* (Atlantic salmon); Sco s, *Scomber scombrus* (Atlantic mackerel); Thu a, *Thunnus albacares* (yellowfin tuna); Tri s, *Triakis semifasciata* (Leopard shark); Xen l, *Xenopus laevis* (African clawed frog); Xen t, *Xenopus tropicalis* (tropical clawed frog).

All major allergens from bony fish are β-parvalbumins. Family members from different bony fish species shown in Figure 4A share sequence identities between 61% (Lep w 1.0101 and Sal s 1.0101) and 82% (Cyp c 1.0101 and Gad m 1.0101), with the highest extent of conservation found within the calcium binding motifs (Figure 3A). No β-parvalbumins have been found in muscle tissue of higher vertebrates and cartilaginous fish. In contrast, α-parvalbumins exist in all vertebrates with a similar degree of sequence conservation as β-parvalbumins (Figure 4B). α-Parvalbumins show low IgE-binding and were identified as minor allergens in chicken meat (38) and rarely consumed meat from frogs and crocodiles (39, 40). IgE-binding to parvalbumins depends on the presence of calcium as shown by reduced IgE-binding after calcium depletion or mutation of the calcium binding sites (41, 42).

A number of studies showed IgE cross-reactivity based on conserved IgE epitopes on β-parvalbumins from different fish species and these are discussed by Klueber et al. (24). In contrast, salmonid fish showed the presence of a species-specific IgE epitope of parvalbumin and the absence of cross-reactivity to other fish (43, 44). It is possible that parvalbumins from other fish families also have specific IgE epitopes which may be relevant in mono- or oligo-sensitized patients. The cross-reactivity between bony fish-derived β- and cartilaginous fish-derived α-parvalbumins was limited in patients primarily sensitized to bony fish (26). IgE cross-reactivity between parvalbumins from fish and other vertebrates was shown between the frog β parvalbumin Ran e 2 and cod parvalbumin (45), between chicken and cod parvalbumins (38), and between crocodile and hake/cod parvalbumins (40). These aspects should be taken into consideration during the clinical workup of the patient and the management plan.

#### β-enolase

Enolase is a glycolytic enzyme found in all organisms that catalyzes the reversible dehydration of 2-phosphoglycerate to phosphoenolpyruvate. A metal ion, Mg^2+^, is needed as a cofactor for the two-step enolase reaction. In vertebrates, the enzyme exists in three tissue-specific isoforms: α, β, and γ (46). Enolases form dimers of which both homo- and heterodimers exist (Figure 3C). The predominant isoform expressed in muscle is the ββ-homodimer. Each monomer consists of two domains (Figure 3C), an N-terminal domain folding into a two-layer sandwich composed of an anti-parallel β-sheet and an α-helical bundle, and a C-terminal domain which folds into a triosephosphate isomerase (TIM)-like α/β-barrel (47).

Fish β-enolases show sequences identities > 80% with their human homolog (Figure 4C), yet they were identified as allergens in several fish species such as cod (Gad m 2), and salmon (Sal s 2) (48), but also in chicken meat (Gal d 9) (38). Sequence identities between enolases from bony fish exceed 90% (Figure 4C). Kuehn et al. analyzed the IgE cross-reactivity between cod, salmon and tuna enolases by IgE inhibition experiments (48). The IgE cross-reactivity was highly variable, the IgE-binding to salmon and tuna being the most efficiently inhibited by cod enolase, possibly due to a primary sensitization to cod among the recruited patients. This variability may be attributed to less conserved patches on the molecular surface despite the high overall conservation (Figure 3C).

#### Aldolase A

Fructose-bisphosphate aldolase, often referred to simply as aldolase, is an essential enzyme in glycolysis and gluconeogenesis which catalyzes the reversible reaction that cleaves the aldol fructose-1,6-bisphosphate into the triose phosphates dihydroxyacetone phosphate (DHAP) and glyceraldehyde 3-phosphate (G3P). Aldolases are divided into two classes. While class II aldolases are less investigated and mainly characterized from prokaryotes and fungi (49), more is known about the class I aldolases which are present in some bacteria, archaea, higher plants, and animals (50). There are three forms of class I aldolases, aldolase A, B, and C. Aldolase isozymes are expressed in a tissue-specific manner only in vertebrates. Aldolase A is preferentially expressed in muscle and brain. The active enzymes are tetramers that consist of subunits folding into TIM α/β-barrels (Figure 3D).

Similar to enolases, aldolases were identified as minor allergens in several fish species such as cod (Gad m 3), salmon (Sal s 3) and tuna (Thu a 3), and in chicken meat (Gal d 10) (38, 48). Kuehn et al. observed the IgE cross-reactivity to aldolases from cod, salmon and tuna among the fish-allergic patients, but the degree of cross-reactivity varied among patients greater than in the case of enolases (48).

#### Tropomyosin

Tropomyosins are α-helical coiled-coil parallel dimers that form head-to-tail polymers along the length of actin filaments (Figure 3E). Tropomyosins regulate morphogenesis, cell proliferation, vesicle trafficking, biomechanics, and glucose metabolism in an isoform-specific manner by orchestrating the interaction of actin filaments with myosin motors and actin-binding proteins (51). The actin-based thin filament is composed of a double-stranded polymer of actin, two continuous polymers of tropomyosin running along each side of the actin and the troponin complex. Muscle contractions involve calcium- and myosin-induced changes in the position of tropomyosin along the actin-based thin filaments to facilitate the engagement of the myosin heads in the thick filament with actins in the thin filament (52).

Tropomyosins from invertebrates are major food allergens in crustaceans and mollusks as well as minor respiratory allergens in mites and cockroaches (53). In contrast, vertebrate tropomyosins show low allergenicity, most likely as a consequence of their high sequence identities with human homologs (Figure 4E) (54). Ore m 4 from tilapia was the first allergenic fish tropomyosin described (55). The purified allergen bound IgE from all ten tested patients with confirmed tilapia allergy and was cross-reactive with shrimp tropomyosin. Four of the ten patients tested in that study were also diagnosed with shrimp allergy. Hence, the primary sensitizing allergen and the clinical relevance of the cross-reactivity between crustacean and fish tropomyosins remains to be clarified. Recently, salmon (Sal s 4) and catfish (Pan h 4) tropomyosins were identified as heat-stable allergens that bound IgE from 6 to 32% of fish-allergic patients' sera, depending on the species and the isoform (56). Although no data on cross-reactivity between tropomyosins from different fish are available it is expected to be high, given the 80–95% sequence identities between these allergens (Figure 4E).

#### Type I Collagen

Collagen is the main structural protein in the extracellular matrix of various connective tissues including cartilage, bones, tendons, and skin. Collagen forms triple helical fibrils known as the collagen helix (57). The collagen superfamily comprises 28 members, also known as collagen types, which are numbered with Roman numerals (58). The triple helix in type I collagen encompasses 96% of its structure while in collagen type XII it is <10%. The three polypeptide chains are called α chains. The classical fibrillar collagen chains can associate into homotrimers, heterotrimers or both as is the case for collagen I (59).

Sensitization to fish gelatin, the denatured and partially hydrolyzed form of type I collagen, was detected in a minority of fish-allergic patients (48, 60). High IgE cross-reactivity was observed between gelatin from different fish species. In contrast, bovine gelatin was not cross-reactive with fish gelatin (60), which can be explained by a considerable sequence divergence between mammalian and fish type I collagens (Figure 4F). Type I collagen extracted from the meat and skin of several fish species bound IgE from 20% of fish-allergic patients and activated basophils sensitized with patients' sera (61).

#### Creatine Kinase

Creatine, which is produced from glycine and arginine with an additional requirement for methionine, is found primarily in muscle and brain tissue of vertebrates. Phosphocreatine is used as a reservoir of high-energy phosphate and serves as a donor of a phosphoryl group to convert adenosine diphosphate (ADP) back to adenosine triphosphate (ATP) during intense activity. Excess ATP is used during a period of low effort to convert creatine back to phosphocreatine. Creatine kinase (CK), a key enzyme of the cellular energy metabolism, catalyzes this conversion of creatine to phosphocreatine. Three dimeric isoforms, ubiquitous brain type BB-CK, sarcomeric muscle type MM-CK and the MB-CK heterodimer (62) and two octameric mitochondrial isoforms (63) are synthesized in a tissue-specific manner. Among the known CK crystal structures, only one from a cartilaginous fish, the Pacific electric ray (*Torpedo californica*) has been determined (64).

CK was identified as a minor allergen in catfish (Pan h 7) and salmon (Sal s 7) by a combination of IgE immunoblotting and mass spectrometric identification of IgE-binding bands (56). In the same study, Ruethers et al. identified several additional fish allergens with biological functions as enzymes in metabolic processes, described below.

#### Triosephosphate Isomerase

Triosephosphate isomerase (TIM), another important enzyme in glycolysis, catalyzes the reversible interconversion of the triose phosphate isomers dihydroxyacetone phosphate and D-glyceraldehyde 3-phosphate. TIM is a perfectly evolved enzyme with a very fast interconversion rate (65). The enzyme, which forms dimers of two identical subunits, is found in bacteria, fungi, plants and animals (66). Ruethers et al. identified TIM as a minor allergen from catfish (Pan h 8) and salmon (Sal s 8) (56).

#### Pyruvate Kinase

Pyruvate kinase (PK) catalyzes the last and irreversible step of glycolysis, the transfer of a phosphate group from phosphoenolpyruvate (PEP) to ADP resulting in the production of pyruvic acid and ATP (67). PK is encoded by two paralogous genes, each of which is alternatively spliced to yield four distinct, tissue-specific isozymes in vertebrates (68). The L and R isozymes, derived from the *PKLR* gene, show tissue-specific expression in the liver and erythrocytes, respectively. The *PKM2* gene consists of 12 exons, of which exons 9 and 10 are alternatively spliced in a mutually exclusive fashion to give rise to the PK-M1 (muscle and brain) and PK-M2 (fetal tissue and adult tissues) isoforms, respectively (69). PK-M from catfish (Pan h 9) was identified as a minor allergen (56).

#### Lactate Dehydrogenase

Lactate dehydrogenase (LDH) catalyzes the reduction of pyruvate, the final product of glycolysis, with NADH to form lactate (70). LDH converts pyruvate to lactate when oxygen is absent or in short supply during strenuous muscular activity. The lactate formed can be recycled in the liver, where it is converted to glucose. Pan h 10 is an LDH from catfish identified by Ruethers et al. as a minor allergen (56).

#### Glucose-6-Phosphate Isomerase

Inside the cell, glucose 6-phosphate isomerase or phosphoglucose isomerase functions as a housekeeping enzyme of glycolysis and gluconeogenesis. It interconverts glucose 6-phosphate and fructose 6-phosphate, a reaction driven solely by the relative proportions of these sugars in the cytosol. In extracellular processes, phosphoglucose isomerase has been linked to a variety of cytokine activities (71). Catfish phosphoglucose isomerase (Pan h 11) was identified as a minor allergen (56).

#### Glyceraldehyde-3-Phosphate Dehydrogenase

Glyceraldehyde 3-phosphate dehydrogenase (GAPDH) catalyzes the sixth step of glycolysis, the conversion of glyceraldehyde 3-phosphate to 1,3-bisphosphoglycerate. GAPDH from catfish (Pan h 13) was shown to be a minor allergen, as described through IgE immunoblotting using fish-allergic patients' sera and mass spectrometric identification of the IgE-binding bands (56).

#### Vitellogenin

Vitellogenins are the major egg yolk proteins and the source of nutrients required for the development of the embryo of egg-laying vertebrates and invertebrates. Vitellogenins, which are produced in the liver, provide or transport amino acids, lipids, phosphorous, and calcium to the egg (72). In addition, vitellogenin and its post-translational cleavage products, lipovitellin and phosvitin, have anti-microbial and anti-oxidant activities (73). Onc k 5, the β'-component, a 19 kDa cleavage product of the vitellogenin from chum salmon, is a major allergen for patients allergic to chum salmon roe and showed IgE-cross-reactivity with homologs from fish roe of other salmonids (74).

### Fish Allergens in Food and Cosmetic Products

Due to the possibility of severe allergic reactions, management of fish allergy is usually based on the complete avoidance of fish and other foods containing fish products. Correct food labeling is hence of immense importance for the safety of affected individuals. For correct labeling, reliable approaches for the detection of fish in food products are crucial. A constant effort of researchers and laboratories worldwide is ongoing to improve and expand the available test products for fish allergen detection and subsequent correct food labeling, and to reduce false positive and false negative outcomes (75).

Several different methods are used for the detection and quantification of allergens in food, such as enzyme-linked immunosorbent assay (ELISA), polymerase chain reaction (PCR), lateral flow devices, and liquid chromatography coupled with mass spectrometry (LC/MS) (76). Lately, besides the traditional analytical methods, novel approaches are considered with the intention to make food allergen detection more user-friendly, such as smartphone-based detection methods (77).

For the detection of fish, DNA-based tests are commercially available from several manufacturers. However, they can only detect a limited number of bony fish species, which restricts their use considering the wide range of species relevant for human consumption. In addition, the available ELISA kits for fish allergen detection are mostly developed to recognize Atlantic cod and its major allergen Gad m 1, which is mainly relevant for Europe and North America. A recent study by Ruethers et al. compared the ability of three commercially available ELISA kits to detect a range of bony and cartilaginous fish (76). The researchers found that, depending on the kit, only 26–61% of the 57 bony fish species tested were detected. In addition, seven cartilaginous fish extracts were analyzed, and none was detected using these kits. These results emphasized the need for improvement of methods for fish detection in food products.

Unexpected fish allergens can also be found in other products. Type I collagen from fish was recently characterized as an allergen with particular biochemical properties (56). It is only found in low quantities in fish extracts prepared in aqueous neutral solutions (61). Besides in food products and supplements (marshmallows, edible films and coatings, drinks etc.), fish collagen and its denatured form gelatin are found in various cosmetic and pharmaceutical products including wound dressings and dental applications (78–80). Inclusion of this allergen in detection kits is therefore urgently required for increased patient safety.

## Food Processing and Its Impact on Fish Allergenicity

Besides the diversity of species and allergens, different fish preparation methods further increase the complexity of fish allergy diagnosis. Various processing treatments are used in the preparation of fish and products thereof to increase the shelf-life, improve the taste, texture, and remove pathogenic microorganisms. Fish allergens belong to several protein families (see above) with different levels of stability to food processing. The food processing techniques may alter allergen recognition by IgE, as they may lead to events such as fragmentation of linear IgE epitopes, destruction of the conformational epitopes, or the generation of neoepitopes. Processes such as heating, smoking or drying resulted in decreased protein solubility and in the denaturation of some proteins. These and other aspects of changes in allergenicity upon fish processing were recently reviewed by Dasanayaka et al. (81) and by Abraha et al. (82).

In addition, dietary habits differ worldwide, and this suggests that different patterns of sensitization to specific fish species and allergens may be expected in different geographic regions. In line with that, diagnostic tests for fish allergy may require optimization regarding local dietary habits and may need to include, besides the allergen components, raw and/or heated fish extracts.

Parvalbumin is the major allergen from fish muscle. Several studies investigated IgE-binding to parvalbumin from fish processed by different methods. IgE reactivity (by Western blot and β-hexosaminidase release by basophils) of the parvalbumin from the Japanese scad (*Decapterus maruadsi*) was retained after boiling, ultraviolet irradiation and ultrasonication, but decreased upon Maillard reaction and pressure treatment (83). Another study examined the thermal stability of Pacific mackerel parvalbumin up to 140°C and found a reduced IgE-binding to the heated protein (84).

Sletten et al. analyzed IgE-binding of fish-allergic patients to several fresh and processed fish species (85). Processed fish were smoked, salted/sugar-cured, canned, lye-treated or fermented. Smoking of fish appeared to increase the parvalbumin IgE-binding, while chemical processing had an opposite effect (85). The differential stability of antibody epitopes on parvalbumin was demonstrated by Liang et al., showing differences of polyclonal and monoclonal antibody binding to heated as compared to non-heated parvalbumin (86). Using polyclonal anti-parvalbumin antibodies, Sharp et al. demonstrated absence of parvalbumin recognition in extracts from canned fish due to protein degradation during the canning process, using four polyclonal anti-parvalbumin IgG antibodies (87).

The same fish usually contains several parvalbumin isoforms, which may differ in their stability and IgE reactivity. Perez-Tavarez et al. investigated the parvalbumin composition from Atlantic cod and chub mackerel and discovered that parvalbumin isoforms with the strongest IgE-binding displayed protease-sensitive globular folds, while the amyloid-forming isoforms were not strongly immunoreactive (88). In the Indian mackerel seven parvalbumin isoforms were identified, demonstrating different IgE-binding capacities and molecular weights ranging from 10.5 to 12.6 kDa (89). Parvalbumin isoform properties from single fish species should be further characterized and considered when deciding on the right isoforms to be used in diagnostic approaches.

Type I collagen is a fish allergen abundant in bones, skin and muscles of fish. While not efficiently extracted at neutral pH and room temperature, collagen can be extracted from fish tissue by acidic treatment, heating or enzymatic treatment (90). Due to the increased extractability at low pH (also present in stomach), it remains to be elucidated whether thermally processed fish is equally or more dangerous for individuals sensitized to this allergen. In terms of the IgE-binding properties, Kobayashi et al. demonstrated that collagen in fish extracts prepared from thermally processed fish (heated to 100, 120, and 140°C before the extract preparation) retained its IgE-binding properties (91). This demonstrates the importance of collagen as an allergen from both raw and heated fish.

An additional allergen from fish is tropomyosin, identified from tilapia, salmon and catfish (55, 56). Tropomyosin is a well-known invertebrate pan-allergen. Despite its conserved structure and high sequence similarity, tropomyosins from different invertebrate species showed different structural stabilities upon heating (92). However, several studies indicated the retention of IgE-binding to tropomyosin after the heat treatment (93–95).

Several heat-labile fish allergens were described, which are hence primarily important when consuming raw fish. Aldolase A and β-enolase were described as allergens from cod, salmon, catfish, tuna and carp (48, 56). Other IgE-binding proteins from fish have been described which have biological roles as enzymes in metabolic processes (56). The clinical importance of these recently discovered allergens is still to be elucidated. Due to the heat-sensitivity, patients sensitized exclusively to these allergens may be able to consume thermally processed fish. For the precise diagnosis of the sensitization to raw fish, extracts and purified allergens may be required.

## Fish Allergy Diagnosis

### Evaluation of Fish-Allergic Patients

Fish allergy prevalence ranges from 0.1 to 0.5%, as assesed by oral food challenges (OFC) (22). The current clinical approaches to fish allergy diagnosis include clinical assessment, skin prick test (SPT), serum specific-IgE testing, and OFC. Clinical symptoms and medical history underpin the likelihood of fish allergy. For an accurate diagnosis, a detailed history of the allergic episode, including time and duration of the reaction, type and quantity of specific fish species, symptom characteristics involving skin, mucous membranes, cardiovascular, respiratory, and neurologic system, and prior history of a similar reaction are essential aspects to diagnose fish allergies (4). A major drawback is that patients may fail to identify the suspected fish species that trigger their allergic symptoms and to provide a detailed medical history. In addition, mislabeling of seafood products is commonly reported, most frequently for fish (96). Identification of the specific fish causing the allergy is important for further management of the disease. It allows clinicians to understand and better characterize the etiology and characteristics of the reaction and decide on further confirmatory allergy testing. Within the large group of fish, most reported allergies are to bony fish, whereas cartilaginous fish (rays and sharks) seem to be of lower allergenicity (26). It is also valuable to consider allergy to shellfish. Although the rates of cross-reactivity between fish and shellfish are not expected to be high due to differences in major allergens, co-allergy to both fish and crustaceans was reported for between 6 and 21% of patients with seafood allergy (97). Moreover, up to 29% of fish-allergic patients report allergy to multiple fish species (98).

SPT is a common screening procedure for IgE-mediated food allergy by examining skin reactivity to food extracts. It is a reliable method for patients to rapidly determine their sensitization results. Although SPT is a sensitive test method for fish allergy, commercially available fish extracts are limited compared to the wide variety of fish species. Prick-to-prick tests using fresh food can circumvent this obstacle. The reliability of SPT is greatly hampered by the lack of allergen standardization, lack of important allergens and the presence of preservatives in the extracts (99).

Serum-specific IgE quantification is a commonly used *in vitro* method to determine the sensitization to specific fish allergens and the potential for clinical reactivity to various fish species. In the widely used ImmunoCAP system (ThermoFisher Phadia), 28 fish extracts but only 2 purified fish allergens are available for routine specific-IgE quantification. The combination of a characteristic clinical history of an allergic reaction against fish with elevated levels of allergen-specific IgE or a positive SPT result is the foundation of fish allergy diagnosis.

Oral food challenge is the only diagnostic *in vivo* test to confirm fish allergy and a positive OFC usually correlates with a strong SPT result and high specific IgE against fish. OFCs can be performed open, single-blind, or DBPCFC, the latter being the gold standard for the diagnosis of fish allergy. OFC is useful not only for the diagnosis but also for avoiding unnecessary dietary restrictions. Several studies used OFC to show that some patients with fish allergy may tolerate certain fish types (5, 26, 100).

### Recommendation for the Clinical Practice and Patient Management

The standard of care for patients with fish allergy remains the dietary avoidance of fish and administration of rescue medicine in case of accidental exposure to fish or their allergens (101, 102). Patients with an allergy to one or more fish are advised to avoid most or all fish species, a recommendation that in many cases turns out to be too strict. It has been shown that patients with fish allergy can frequently eat certain fish species. Therefore, it has been proposed to categorize patients with fish allergy into three clusters: (A) polysensitized patients who respond to all types of fish on the basis of cross-reactions of β-parvalbumin and often enolase and aldolase, (B) mono-sensitized patients with a selective allergic reaction to one individual fish species based on a specific epitope of β-parvalbumin, and (C) oligo-sensitized patients who respond to a number of specific fish based on enolase and aldolase, without IgE for β-parvalbumin (21). Depending on the specific cluster the patient belongs to, OFC for one or more fish species can be performed with the aim to provide safe alternatives for consumption. Following this procedure, several alternative fish species can be usually identified for mono- and oligo-sensitized patients that can safely be consumed. The majority of patients are aware that fish is a valuable source of healthy nutrients and they usually do want to know whether they can consume certain fish species safely.

Data on the natural history of fish allergy are scarce, with the majority of reports suggesting long-term clinical reactivity (103). One study showed that only 15% of children outgrow their fish allergy within a period of 2 to 5 years (104). It has been recently shown in a prospective clinical study that tolerance of fish increased with age, ranging from 3.4% in preschool children to over 45% in adolescents (105). Tolerance of fish was defined by a negative OFC to cod, whereas a positive outcome to any fish challenge or a specific IgE value to cod (f3) > 20 kU/L was defined as an active fish allergy. These results are in line with data from a retrospective study, showing that up to 63% of patients may overcome fish allergy (106). These findings are clinically relevant. Patients developing tolerance over time should be identified to avoid unnecessary continuation of food restrictions.

### Recent Developments in Diagnostic Approaches

A need for diagnostic tests which allow to examine the allergic sensitization to multiple fish species is becoming increasingly recognized. In terms of whole fish extracts, while those prepared from raw fish may contain native heat-sensitive allergens, heating of the extracts may yield a certain amount of fish collagen. Therefore, both ways of extract preparation should be considered and can be tailored to individual needs of patients or geographic regions, depending on dietary habits.

Molecular allergy diagnosis utilizes purified natural or recombinant allergens instead of total extracts (101). In addition to easy standardization, this approach may be very useful for fish allergy diagnosis as it allows for simultaneous quantification of IgE specific to many species and may therefore reduce the number of required OFCs (4, 107). Besides the major allergen parvalbumin, other allergen components should be implemented to precisely determine the pattern of IgE sensitization. One example of a recently introduced *in vitro* multiplex allergen test is the ALEX2 Allergy Explorer (MacroArray Diagnostics). It contains, in terms of fish allergens, parvalbumins from 8 species, as well as aldolase and enolase from Atlantic cod, and whole extracts from 6 fish species. This is the first commercially available test which includes total extracts from allergen sources as well as an α parvalbumin from thornback ray, a species which may be tolerated by some patients with fish allergy.

Although such multiplex tests can indicate which species the patients are sensitized to and which they may tolerate, OFCs are still required to definitely rule out the reactivity to some species. OFC using purified natural allergens may be an attractive alternative to OFC using total fish extracts. While the methods to purify these allergens are widely available, regulatory aspects and standardization issues make this approach highly unlikely in the near future. Moreover, the presence of specific IgE does not always translate into a clinical reactivity and further research is required to determine the usefulness of multiple IgE testing in fish allergy.

Besides the common approaches used in the clinical setting such as specific IgE quantification, the basophil activation test (BAT) may be a safe alternative to OFCs (108). This assay demonstrated superior specificity to *in vitro* IgE tests for peanut allergy and was also successfully utilized to diagnose food allergy to other sources including egg, milk, hazelnut and wheat (108, 109).

In fish allergy research, BAT using purified fish parvalbumins was efficiently used to identify patients with confirmed tolerance of thornback ray (26). Imakiire et al. used BAT and the ROC analysis to predict the reactivity of fish-allergic patients to extracts from 15 fish species and found a diagnostic accuracy of at least 0.6 when a minimum of five patients were tested with the specific extract (scale for diagnostic accuracy was 0.0–1.0) (110). Further clinical validation of the usefulness of BAT to identify the potentially tolerated species during the diagnosis of fish-allergic patients should be performed in the future.

In addition to BAT, the newly described mast-cell activation test (MAT) should also be investigated for its potential use in fish allergy diagnosis (111). Bahri et al. developed and validated this test for diagnosis of peanut allergy in 2018 but the test has not yet been investigated for fish allergy.

## Conclusions

Precision medicine will play a key role in the management of allergic patients by providing tools when devising treatment options and offering specific guidelines to groups of afflicted individuals. In order to improve the quality of life of fish-allergic individuals, there is a strong need to replace the common practice of advising them to generally avoid all fish by more specific advice. Such advice will have to be based on a comprehensive molecular diagnosis employing a wide range of species-specific allergens. These allergens will have to be selected from related fish species and, more importantly, from fish species that are only distantly related and thus cover the extent of the phylogenetic tree of fishes. Secondly, these allergens will have to reflect the exposure to fish species available in various geographic regions. In practice, the ideal allergen microarray would include sets of parvalbumins and other relevant fish allergens to allow a precise diagnosis of each individual patient. The test result would then have to be confirmed by controlled exposure which ideally would point out options for an individual's diet; taking into account which species are actually available for any given patient.

While allergy to fish is considered to be a long-lasting allergy, there is growing evidence that a considerable portion of fish-allergic children will outgrow their allergic reactivity to fish proteins. In addition, there have been reports of adult patients who became tolerant to fish after strict avoidance. A new evaluation of who continues to be allergic and who can again tolerate several fish species should be offered to fish-allergic patients from time to time using the above-mentioned modern tools for molecular allergy diagnosis and additional OFCs. This would markedly enhance the quality of life for an individual patient and would help to avoid unnecessary dietary restrictions. The finding that long-lived memory B cells can be reactivated after allergen exposure and regenerate allergen-specific IgE in mouse experiments indicates that an OFC might have the same potential (112). It seems therefore advisable to measure specific IgE levels after performing an OFC. The clinical relevance of increased specific IgE after an OFC with fish extracts has yet to be determined in a prospective clinical study.

While parvalbumins remain the most relevant fish allergens, recent years have seen the emergence of a number of new fish allergens. The WHO/IUIS Allergen Nomenclature Sub-Committee (http://www.allergen.org) currently lists 12 types of fish allergens (Table 2). Many of the newly discovered fish allergens are metabolic enzymes, 7 of which are involved in glycolysis. Creatine kinase is a key enzyme of the cellular energy metabolism. Tropomyosin is an integral part of the cytoskeleton, collagen is a structural protein, and vitellogenin is the major yolk protein in fish roe. Enzymes of the glycolytic pathway are well-conserved in evolution. Hence, the respective fish allergens all possess high identities to their human homologs. Whether these proteins are full-fledged allergens or the production of specific IgE is a bystander effect will have to be studied in more detail.

## Author Contributions

TK designed the manuscript layout. TK and CR prepared figures and tables. All authors contributed to manuscript writing, its critical reviewing and approved the final version.

## Funding

Funding for this work was provided by the Danube Allergy Research Cluster of Lower Austria (P-06), the Medical University of Vienna, the Austria Science Fund (FWF) grant P 30936-B30, and the NHMRC grant APP1086656. The authors acknowledge the support by the Open Access Publishing Fund of the Karl Landsteiner University of Health Sciences, Krems, Austria.

## Conflict of Interest

The authors declare that the research was conducted in the absence of any commercial or financial relationships that could be construed as a potential conflict of interest.

## Publisher's Note

All claims expressed in this article are solely those of the authors and do not necessarily represent those of their affiliated organizations, or those of the publisher, the editors and the reviewers. Any product that may be evaluated in this article, or claim that may be made by its manufacturer, is not guaranteed or endorsed by the publisher.

## Abbreviations

ATP: adenosine triphosphate
BAT: basophil activation test
CK: creatine kinase
DBPCFC: double-blind placebo-controlled food challenge
EAACI: European Academy of Allergy and Clinical Immunology
ELISA: enzyme-linked immunosorbent assay
FAO: Food and Agriculture Organization of the United Nations
FPIAP: food protein induced allergic proctocolitis
GAPDH: glyceraldehyde 3-phosphate dehydrogenase
LC/MS: liquid chromatography coupled with mass spectrometry
LDH: lactate dehydrogenase
OFC: oral food challenge
PCR: polymerase chain reaction
PK: pyruvate kinase
SPT: skin prick test
TIM: triosephosphate isomerase.

## References

[1] Johansson SG Hourihane JO Bousquet J Bruijnzeel-Koomen C Dreborg S Haahtela T. A revised nomenclature for allergy. An EAACI position statement from the EAACI nomenclature task force. Allergy. (2001) 56:813–24. 10.1034/j.1398-9995.2001.t01-1-00001.x 11551246

[2] Connors L O'Keefe A Rosenfield L Kim H. Non-IgE-mediated food hypersensitivity. Allergy Asthma Clin Immunol. (2018) 14 (Suppl. 2):56. 10.1186/s13223-018-0285-2 30275846 PMC6157279

[3] Buyuktiryaki B Masini M Mori F Barni S Liccioli G Sarti L. IgE-mediated fish allergy in children. Medicina. (2021) 57:76. 10.3390/medicina57010076 33477460 PMC7830012

[4] Davis CM Gupta RS Aktas ON Diaz V Kamath SD Lopata AL. Clinical management of seafood allergy. J Allergy Clin Immunol Pract. (2020) 8:37–44. 10.1016/j.jaip.2019.10.019 31950908

[5] Sørensen M Kuehn A Mills ENC Costello CA Ollert M Småbrekke L. Cross-reactivity in fish allergy: a double-blind, placebo-controlled food-challenge trial. J Allergy Clin Immunol. (2017) 140:1170–2. 10.1016/j.jaci.2017.03.043 28479332

[6] Belton B Thilsted SH. Fisheries in transition: food and nutrition security implications for the global South. Global Food Security. (2014) 3:59–66. 10.1016/j.gfs.2013.10.001

[7] Gupta RS Springston EE Warrier MR Smith B Kumar R Pongracic J. The prevalence, severity, and distribution of childhood food allergy in the United States. Pediatrics. (2011) 128:e9–17. 10.1542/peds.2011-0204 21690110

[8] Gupta RS Warren CM Smith BM Jiang J Blumenstock JA Davis MM. Prevalence and severity of food allergies among US adults. JAMA Netw Open. (2019) 2:e185630. 10.1001/jamanetworkopen.2018.5630 30646188 PMC6324316

[9] Chen J Liao Y Zhang HZ Zhao H Chen J Li HQ. Prevalence of food allergy in children under 2 years of age in three cities in China. Zhonghua Er Ke Za Zhi. (2012) 50:5–9. 10.1097/01.WOX.0000411608.35185.f5 22456067

[10] Dalal I Binson I Reifen R Amitai Z Shohat T Rahmani S. Food allergy is a matter of geography after all: sesame as a major cause of severe IgE-mediated food allergic reactions among infants and young children in Israel. Allergy. (2002) 57:362–5. 10.1034/j.1398-9995.2002.1s3412.x 11906370

[11] Connett GJ Gerez I Cabrera-Morales EA Yuenyongviwat A Ngamphaiboon J Chatchatee P. A population-based study of fish allergy in the Philippines, Singapore and Thailand. Int Arch Allergy Immunol. (2012) 159:384–90. 10.1159/000338940 22846665

[12] Al-Hammadi S Al-Maskari F Bernsen R. Prevalence of food allergy among children in Al-Ain city, United Arab Emirates. Int Arch Allergy Immunol. (2010) 151:336–42. 10.1159/000250442 19851075

[13] Le TTK Nguyen DH Vu ATL Ruethers T Taki AC Lopata AL. A cross-sectional, population-based study on the prevalence of food allergies among children in two different socio-economic regions of Vietnam. Pediatr Allergy Immunol. (2019) 30:348–55. 10.1111/pai.13022 30793379

[14] Obeng BB Amoah AS Larbi IA Yazdanbakhsh M van Ree R Boakye DA. Food allergy in Ghanaian schoolchildren: data on sensitization and reported food allergy. Int Arch Allergy Immunol. (2011) 155:63–73. 10.1159/000318704 21109750

[15] Pyrhönen K Näyhä S Kaila M Hiltunen L Läärä E. Occurrence of parent-reported food hypersensitivities and food allergies among children aged 1-4 yr. Pediatr Allergy Immunol. (2009) 20:328–38. 10.1111/j.1399-3038.2008.00792.x 19538354

[16] Pénard-Morand C Raherison C Kopferschmitt C Caillaud D Lavaud F Charpin D. Prevalence of food allergy and its relationship to asthma and allergic rhinitis in schoolchildren. Allergy. (2005) 60:1165–71. 10.1111/j.1398-9995.2005.00860.x 16076302

[17] Grabenhenrich L Trendelenburg V Bellach J Yürek S Reich A Fiandor A. Frequency of food allergy in school-aged children in eight European countries-The EuroPrevall-iFAAM birth cohort. Allergy. (2020) 75:2294–308. 10.1111/all.14290 32219884

[18] Schnabel E Sausenthaler S Schaaf B Schäfer T Lehmann I Behrendt H. Prospective association between food sensitization and food allergy: results of the LISA birth cohort study. Clin Exp Allergy. (2010) 40:450–7. 10.1111/j.1365-2222.2009.03400.x 19958366

[19] Eggesbø M Halvorsen R Tambs K Botten G. Prevalence of parentally perceived adverse reactions to food in young children. Pediatr Allergy Immunol. (1999) 10:122–32. 10.1034/j.1399-3038.1999.00022.x 10478614

[20] Ostblom E Lilja G Pershagen G van Hage M Wickman M. Phenotypes of food hypersensitivity and development of allergic diseases during the first 8 years of life. Clin Exp Allergy. (2008) 38:1325–32. 10.1111/j.1365-2222.2008.03010.x 18477012

[21] Dijkema D Emons JAM Van de Ven A Oude Elberink JNG. Fish allergy: fishing for novel diagnostic and therapeutic options. Clin Rev Allergy Immunol. (2020). 10.1007/s12016-020-08806-5. [Epub ahead of print]. 32712803 PMC8818006

[22] Ruethers T Taki AC Johnston EB Nugraha R Le TTK Kalic T. Seafood allergy: a comprehensive review of fish and shellfish allergens. Mol Immunol. (2018) 100:28–57. 10.1016/j.molimm.2018.04.008 29858102

[23] Le TTK Tran TTB Ho HTM Vu ATL McBryde E Lopata AL. The predominance of seafood allergy in Vietnamese adults: results from the first population-based questionnaire survey. World Allergy Organ J. (2020) 13:100102. 10.1016/j.waojou.2020.100102 32161634 PMC7058921

[24] Klueber J Schrama D Rodrigues P Dickel H Kuehn A. Fish allergy management: from component-resolved diagnosis to unmet diagnostic needs. Curr Treat Options Allergy. (2019) 6:322–37. 10.1007/s40521-019-00235-w

[25] Stephen JN Sharp MF Ruethers T Taki A Campbell DE Lopata AL. Allergenicity of bony and cartilaginous fish - molecular and immunological properties. Clin Exp Allergy. (2017) 47:300–12. 10.1111/cea.12892 28117510

[26] Kalic T Morel-Codreanu F Radauer C Ruethers T Taki AC Swoboda I. Patients allergic to fish tolerate ray based on the low allergenicity of its parvalbumin. J Allergy Clin Immunol Pract. (2019) 7:500–8. 10.1016/j.jaip.2018.11.011 30471362 PMC7060078

[27] FAO. The State of World Fisheries and Aquaculture 2020. Sustainability in Action. Rome, Italy: FAO (2020).

[28] FAO Needham S Funge-Smith S. The Consumption of Fish and Fish Products in the Asia-Pacific Region Based on Household Surveys. Bangkok: FAO (2015).

[29] Szucs I Tikász IE Fehér M Stündl L. Testing for consumer preferences of smoked asian sea bass (Barramundi) filet products in Hungary. Cogent Bus Manage. (2018) 5:1432158. 10.1080/23311975.2018.1432158

[30] Shamshak GL Anderson JL Asche F Garlock T Love DC. U.S. seafood consumption. J World Aquacult Soc. (2019) 50:715–27. 10.1111/jwas.12619

[31] Liang J Tan CC Taylor SL Baumert JL Lopata AL Lee NA. Quantitative analysis of species specificity of two anti-parvalbumin antibodies for detecting southern hemisphere fish species demonstrating strong phylogenetic association. Food Chem. (2017) 237:588–96. 10.1016/j.foodchem.2017.05.153 28764040

[32] Bugajska-Schretter A Elfman L Fuchs T Kapiotis S Rumpold H Valenta R. Parvalbumin, a cross-reactive fish allergen, contains IgE-binding epitopes sensitive to periodate treatment and Ca2+ depletion. J Allergy Clin Immunol. (1998) 101:67–74. 10.1016/S0091-6749(98)70195-2 9449503

[33] Van Do T Hordvik I Endresen C Elsayed S. The major allergen (parvalbumin) of codfish is encoded by at least two isotypic genes: cDNA cloning, expression and antibody binding of the recombinant allergens. Mol Immunol. (2003) 39:595–602. 10.1016/S0161-5890(02)00200-6 12431393

[34] Nockolds CE Kretsinger RH Coffee CJ Bradshaw RA. Structure of a calcium-binding carp myogen. Proc Natl Acad Sci USA. (1972) 69:581–4. 10.1073/pnas.69.3.581 4501574 PMC426511

[35] Arif SH. A Ca(2+)-binding protein with numerous roles and uses: parvalbumin in molecular biology and physiology. Bioessays. (2009) 31:410–21. 10.1002/bies.200800170 19274659

[36] Climer LK Cox AM Reynolds TJ Simmons DD. Oncomodulin: the enigmatic parvalbumin protein. Front Mol Neurosci. (2019) 12:235. 10.3389/fnmol.2019.00235 31649505 PMC6794386

[37] Larkin MA Blackshields G Brown NP Chenna R McGettigan PA McWilliam H. Clustal W and Clustal X version 2.0. Bioinformatics. (2007) 23:2947–8. 10.1093/bioinformatics/btm404 17846036

[38] Kuehn A Codreanu-Morel F Lehners-Weber C Doyen V Gomez-André SA Bienvenu F. Cross-reactivity to fish and chicken meat - a new clinical syndrome. Allergy. (2016) 71:1772–81. 10.1111/all.12968 27344988

[39] Hilger C Grigioni F Thill L Mertens L Hentges F. Severe IgE-mediated anaphylaxis following consumption of fried frog legs: definition of alpha-parvalbumin as the allergen in cause. Allergy. (2002) 57:1053–8. 10.1034/j.1398-9995.2002.23677.x 12359003

[40] Haroun-Díaz E Blanca-López N Vázquez de la Torre M Ruano FJ Somoza Álvarez ML Labrador Horrillo M. Severe anaphylaxis due to crocodile-meat allergy exhibiting wide cross-reactivity with fish allergens. J Allergy Clin Immunol Pract. (2018) 6:669–70. 10.1016/j.jaip.2017.07.015 28923489

[41] Bugajska-Schretter A Grote M Vangelista L Valent P Sperr WR Rumpold H. Purification, biochemical, and immunological characterisation of a major food allergen: different immunoglobulin E recognition of the apo- and calcium-bound forms of carp parvalbumin. Gut. (2000) 46:661–9. 10.1136/gut.46.5.661 10764710 PMC1727915

[42] Zuidmeer-Jongejan L Huber H Swoboda I Rigby N Versteeg SA Jensen BM. Development of a hypoallergenic recombinant parvalbumin for first-in-man subcutaneous immunotherapy of fish allergy. Int Arch Allergy Immunol. (2015) 166:41–51. 10.1159/000371657 25765512

[43] Kuehn A Hutt-Kempf E Hilger C Hentges F. Clinical monosensitivity to salmonid fish linked to specific IgE-epitopes on salmon and trout beta-parvalbumins. Allergy. (2011) 66:299–301. 10.1111/j.1398-9995.2010.02463.x 20804469

[44] Peñas E Uberti F Baviera G Di Lorenzo C Restani P. Clinical monosensitivity to salmon and rainbow trout: a case report. Pediatr Allergy Immunol. (2014) 25:98–100. 10.1111/pai.12150 24237010

[45] Hilger C Thill L Grigioni F Lehners C Falagiani P Ferrara A. IgE antibodies of fish allergic patients cross-react with frog parvalbumin. Allergy. (2004) 59:653–60. 10.1111/j.1398-9995.2004.00436.x 15147451

[46] Piast M Kustrzeba-Wójcicka I Matusiewicz M Banaś T. Molecular evolution of enolase. Acta Biochim Pol. (2005) 52:507–13. 10.18388/abp.2005_3466 15912209

[47] Livesay D La D. The evolutionary origins and catalytic importance of conserved electrostatic networks within TIm barrel proteins. Prot Sci. (2005) 14:1158–1170. 10.1110/ps.041221105 15840824 PMC2253277

[48] Kuehn A Hilger C Lehners-Weber C Codreanu-Morel F Morisset M Metz-Favre C. Identification of enolases and aldolases as important fish allergens in cod, salmon and tuna: component resolved diagnosis using parvalbumin and the new allergens. Clin Exp Allergy. (2013) 43:811–22. 10.1111/cea.12117 23786287

[49] Cooper SJ Leonard GA McSweeney SM Thompson AW Naismith JH Qamar S. The crystal structure of a class II fructose-1,6-bisphosphate aldolase shows a novel binuclear metal-binding active site embedded in a familiar fold. Structure. (1996) 4:1303–15. 10.1016/S0969-2126(96)00138-4 8939754

[50] Tolan DR Niclas J Bruce BD Lebo RV. Evolutionary implications of the human aldolase-A, -B, -C, and -pseudogene chromosome locations. Am J Hum Genet. (1987) 41:907–24. 3674018 PMC1684339

[51] Gunning PW Hardeman EC Lappalainen P Mulvihill DP. Tropomyosin - master regulator of actin filament function in the cytoskeleton. J Cell Sci. (2015) 128:2965–74. 10.1242/jcs.172502 26240174

[52] Lehman W Craig R. Tropomyosin and the steric mechanism of muscle regulation. Adv Exp Med Biol. (2008) 644:95–109. 10.1007/978-0-387-85766-4_8 19209816

[53] Wong L Tham EH Lee BW. An update on shellfish allergy. Curr Opin Allergy Clin Immunol. (2019) 19:236–42. 10.1097/ACI.0000000000000532 30893087

[54] Jenkins JA Breiteneder H Mills EN. Evolutionary distance from human homologs reflects allergenicity of animal food proteins. J Allergy Clin Immunol. (2007) 120:1399–405. 10.1016/j.jaci.2007.08.019 17935767

[55] Liu R Holck AL Yang E Liu C Xue W. Tropomyosin from tilapia (Oreochromis mossambicus) as an allergen. Clin Exp Allergy. (2013) 43:365–77. 10.1111/cea.12056 23414545

[56] Ruethers T Taki AC Karnaneedi S Nie S Kalic T Dai D. Expanding the allergen repertoire of salmon and catfish. Allergy. (2021) 76:1443–53. 10.1111/all.14574 32860256

[57] Brodsky B Persikov AV. Molecular structure of the collagen triple helix. Adv Protein Chem. (2005) 70:301–39. 10.1016/S0065-3233(05)70009-7 15837519

[58] Ricard-Blum S. The collagen family. Cold Spring Harb Perspect Biol. (2011) 3:a004978. 10.1101/cshperspect.a004978 21421911 PMC3003457

[59] Ricard-Blum S Ruggiero F. The collagen superfamily: from the extracellular matrix to the cell membrane. Pathol Biol. (2005) 53:430–42. 10.1016/j.patbio.2004.12.024 16085121

[60] Sakaguchi M Toda M Ebihara T Irie S Hori H Imai A. IgE antibody to fish gelatin (type I collagen) in patients with fish allergy. J Allergy Clin Immunol. (2000) 106:579–84. 10.1067/mai.2000.108499 10984381

[61] Kalic T Kamath SD Ruethers T Taki AC Nugraha R Le TTK. Collagen-an important fish allergen for improved diagnosis. J Allergy Clin Immunol Pract. (2020) 8:3084–92. 10.1016/j.jaip.2020.04.063 32389794

[62] Eppenberger HM Dawson DM Kaplan NO. The comparative enzymology of creatine kinases. I. Isolation and characterization from chicken and rabbit tissues. J Biol Chem. (1967) 242:204–9. 10.1016/S0021-9258(19)81449-7 6016604

[63] Schlegel J Zurbriggen B Wegmann G Wyss M Eppenberger HM Wallimann T. Native mitochondrial creatine kinase forms octameric structures. I. Isolation of two interconvertible mitochondrial creatine kinase forms, dimeric and octameric mitochondrial creatine kinase: characterization, localization, and structure-function relationships. J Biol Chem. (1988) 263:16942–53. 10.1016/S0021-9258(18)37482-9 3182823

[64] Lahiri SD Wang PF Babbitt PC McLeish MJ Kenyon GL Allen KN. The 2.1 A structure of Torpedo californica creatine kinase complexed with the ADP-Mg(2+)-NO(3)(-)-creatine transition-state analogue complex. Biochemistry. (2002) 41:13861–7. 10.1021/bi026655p 12437342

[65] Wierenga RK Kapetaniou EG Venkatesan R. Triosephosphate isomerase: a highly evolved biocatalyst. Cell Mol Life Sci. (2010) 67:3961–82. 10.1007/s00018-010-0473-9 20694739 PMC11115733

[66] Lolis E Petsko GA. Crystallographic analysis of the complex between triosephosphate isomerase and 2-phosphoglycolate at 2.5-A resolution: implications for catalysis. Biochemistry. (1990) 29:6619–25. 10.1021/bi00480a010 2204418

[67] Schormann N Hayden KL Lee P Banerjee S Chattopadhyay D. An overview of structure, function, and regulation of pyruvate kinases. Protein Sci. (2019) 28:1771–84. 10.1002/pro.3691 31342570 PMC6739817

[68] Clower CV Chatterjee D Wang Z Cantley LC Vander Heiden MG Krainer AR. The alternative splicing repressors hnRNP A1/A2 and PTB influence pyruvate kinase isoform expression and cell metabolism. Proc Natl Acad Sci USA. (2010) 107:1894–9. 10.1073/pnas.0914845107 20133837 PMC2838216

[69] Noguchi T Inoue H Tanaka T. The M1- and M2-type isozymes of rat pyruvate kinase are produced from the same gene by alternative RNA splicing. J Biol Chem. (1986) 261:13807–12. 10.1016/S0021-9258(18)67091-7 3020052

[70] Valvona CJ Fillmore HL Nunn PB Pilkington GJ. The regulation and function of lactate dehydrogenase a: therapeutic potential in brain tumor. Brain Pathol. (2016) 26:3–17. 10.1111/bpa.12299 26269128 PMC8029296

[71] Read J Pearce J Li X Muirhead H Chirgwin J Davies C. The crystal structure of human phosphoglucose isomerase at 1.6 A resolution: implications for catalytic mechanism, cytokine activity and haemolytic anaemia. J Mol Biol. (2001) 309:447–63. 10.1006/jmbi.2001.4680 11371164

[72] Brawand D Wahli W Kaessmann H. Loss of egg yolk genes in mammals and the origin of lactation and placentation. PLoS Biol. (2008) 6:e63. 10.1371/journal.pbio.0060063 18351802 PMC2267819

[73] Sun C Zhang S. Immune-Relevant and antioxidant activities of vitellogenin and yolk proteins in fish. Nutrients. (2015) 7:8818–29. 10.3390/nu7105432 26506386 PMC4632452

[74] Shimizu Y Nakamura A Kishimura H Hara A Watanabe K Saeki H. Major allergen and its IgE cross-reactivity among salmonid fish roe allergy. J Agric Food Chem. (2009) 57:2314–9. 10.1021/jf8031759 19226142

[75] Herrero B Vieites JM Espiñeira M. Development of an in-house fast real-time PCR method for detection of fish allergen in foods and comparison with a commercial kit. Food Chem. (2014) 151:415–20. 10.1016/j.foodchem.2013.11.042 24423551

[76] Ruethers T Taki AC Khangurha J Roberts J Buddhadasa S Clarke D. Commercial fish ELISA kits have a limited capacity to detect different fish species and their products. J Sci Food Agric. (2020) 100:4353–63. 10.1002/jsfa.10451 32356561

[77] Ross GMS Bremer M Nielen MWF. Consumer-friendly food allergen detection: moving towards smartphone-based immunoassays. Anal Bioanal Chem. (2018) 410:5353–71. 10.1007/s00216-018-0989-7 29582120 PMC6096701

[78] Kuehn A Hilger C Hentges F. Anaphylaxis provoked by ingestion of marshmallows containing fish gelatin. J Allergy Clin Immunol. (2009) 123:708–9. 10.1016/j.jaci.2008.12.012 19178936

[79] Ueno R Takaoka Y Shimojo N Ohno F Yamaguchi T Matsunaga K. A case of pediatric anaphylaxis caused by gummy tablets containing fish collagen. Asia Pac Allergy. (2020) 10:e35. 10.5415/apallergy.2020.10.e35 33178560 PMC7610083

[80] Subhan F Hussain Z Tauseef I Shehzad A Wahid F. A review on recent advances and applications of fish collagen. Crit Rev Food Sci Nutr. (2021) 61:1027–37. 10.1080/10408398.2020.1751585 32345036

[81] Dasanayaka BP Li Z Pramod SN Chen Y Khan MU Lin H. A review on food processing and preparation methods for altering fish allergenicity. Crit Rev Food Sci Nutr. (2020). 10.1080/10408398.2020.1848791. [Epub ahead of print]. 33307772

[82] Abraha B Admassu H Mahmud A Tsighe N Shui X Fang Y. Effect of processing methods on nutritional and physico-chemical composition of fish: a review. MOJ Food Process Technol. (2018) 6:1. 10.15406/mojfpt.2018.06.00191

[83] Yang H Min J Han XY Li XY Hu JW Liu H. Reduction of the histamine content and immunoreactivity of parvalbumin in Decapterus maruadsi by a Maillard reaction combined with pressure treatment. Food Funct. (2018) 9:4897–905. 10.1039/C8FO01167B 30168566

[84] Kubota H Kobayashi A Kobayashi Y Shiomi K Hamada-Sato N. Reduction in IgE reactivity of Pacific mackerel parvalbumin by heat treatment. Food Chem. (2016) 206:78–84. 10.1016/j.foodchem.2016.03.043 27041301

[85] Sletten G Van Do T Lindvik H Egaas E Florvaag E. Effects of industrial processing on the immunogenicity of commonly ingested fish species. Int Arch Allergy Immunol. (2010) 151:223–36. 10.1159/000242360 19786803

[86] Liang J Taylor SL Baumert J Lopata AL Lee NA. Effects of thermal treatment on the immunoreactivity and quantification of parvalbumin from Southern hemisphere fish species with two anti-parvalbumin antibodies. Food Control. (2021) 121:107675. 10.1016/j.foodcont.2020.107675

[87] Sharp MF Stephen JN Kraft L Weiss T Kamath SD Lopata AL. Immunological cross-reactivity between four distant parvalbumins-Impact on allergen detection and diagnostics. Mol Immunol. (2015) 63:437–48. 10.1016/j.molimm.2014.09.019 25451973

[88] Pérez-Tavarez R Carrera M Pedrosa M Quirce S Rodríguez-Pérez R Gasset M. Reconstruction of fish allergenicity from the content and structural traits of the component β-parvalbumin isoforms. Sci Rep. (2019) 9:16298. 10.1038/s41598-019-52801-6 31704988 PMC6841720

[89] Ruethers T Raith M Sharp MF Koeberl M Stephen JN Nugraha R. Characterization of Ras k 1 a novel major allergen in Indian mackerel and identification of parvalbumin as the major fish allergen in 33 Asia-Pacific fish species. Clin Exp Allergy. (2018) 48:452–63. 10.1111/cea.13069 29193486

[90] Jafari H Lista A Siekapen MM Ghaffari-Bohlouli P Nie L Alimoradi H. Fish collagen: extraction, characterization, and applications for biomaterials engineering. Polymers. (2020) 12:2230. 10.3390/polym12102230 32998331 PMC7601392

[91] Kobayashi Y Kuriyama T Nakagawara R Aihara M Hamada-Sato N. Allergy to fish collagen: Thermostability of collagen and IgE reactivity of patients' sera with extracts of 11 species of bony and cartilaginous fish. Allergol Int. (2016) 65:450–8. 10.1016/j.alit.2016.04.012 27236375

[92] Kamath SD Scheiblhofer S Johnson CM Machado Y McLean T Taki AC. Effect of structural stability on endolysosomal degradation and T-cell reactivity of major shrimp allergen tropomyosin. Allergy. (2020) 75:2909–19. 10.1111/all.14410 32436591 PMC7687109

[93] Rolland JM Varese NP Abramovitch JB Anania J Nugraha R Kamath S. Effect of heat processing on ige reactivity and cross-reactivity of tropomyosin and other allergens of Asia-Pacific mollusc species: identification of novel sydney rock oyster tropomyosin sac g 1. Mol Nutr Food Res. (2018) 62:e1800148. 10.1002/mnfr.201800148 29756679 PMC6099307

[94] James JK Pike DH Khan IJ Nanda V. Structural and dynamic properties of allergen and non-allergen forms of tropomyosin. Structure. (2018) 26:997–1006. 10.1016/j.str.2018.05.002 29887498 PMC6697176

[95] Liu M Huan F Li M Han T Xia F Yang Y. Mapping and IgE-binding capacity analysis of heat/digested stable epitopes of mud crab allergens. Food Chem. (2021) 344:128735. 10.1016/j.foodchem.2020.128735 33279350

[96] Willette DA Simmonds SE Cheng SH Esteves S Kane TL Nuetzel H. Using DNA barcoding to track seafood mislabeling in Los Angeles restaurants. Conserv Biol. (2017) 31:1076–85. 10.1111/cobi.12888 28075039

[97] Cox AL Eigenmann PA Sicherer SH. Clinical relevance of cross-reactivity in food allergy. J Allergy Clin Immunol Pract. (2021) 9:82–99. 10.1016/j.jaip.2020.09.030 33429724

[98] Khan F Orson F Ogawa Y Parker C Davis CM. Adult seafood allergy in the Texas Medical Center: a 13-year experience. Allergy Rhinol. (2011) 2:e71–7. 10.2500/ar.2011.2.0019 22852122 PMC3390121

[99] Ruethers T Taki AC Nugraha R Cao TT Koeberl M Kamath SD. Variability of allergens in commercial fish extracts for skin prick testing. Allergy. (2019) 74:1352–63. 10.1111/all.13748 30762884

[100] Mourad AA Bahna SL. Fish-allergic patients may be able to eat fish. Expert Rev Clin Immunol. (2015) 11:419–30. 10.1586/1744666X.2015.1009896 25666551

[101] Muraro A Werfel T Hoffmann-Sommergruber K Roberts G Beyer K Bindslev-Jensen C. EAACI food allergy and anaphylaxis guidelines: diagnosis and management of food allergy. Allergy. (2014) 69:1008–25. 10.1111/all.12429 24909706

[102] Kourani E Corazza F Michel O Doyen V. What do we know about fish allergy at the end of the decade? J Investig Allergol Clin Immunol. (2019) 29:414–21. 10.18176/jiaci.0381 30741635

[103] Savage J Sicherer S Wood R. The natural history of food allergy. J Allergy Clin Immunol Pract. (2016) 4:196–203. 10.1016/j.jaip.2015.11.024 26968958

[104] Dannaeus A Inganäs M. A follow-up study of children with food allergy. Clinical course in relation to serum IgE- and IgG-antibody levels to milk, egg and fish. Clin Allergy. (1981) 11:533–9. 10.1111/j.1365-2222.1981.tb02171.x 7332999

[105] Xepapadaki P Christopoulou G Stavroulakis G Freidl R Linhart B Zuidmeer L. Natural history of IgE-mediated fish allergy in children. J Allergy Clin Immunol Pract. (2021) 9:3147–56. 10.1016/j.jaip.2021.04.007 33866031

[106] Carvalho S Marcelino J Cabral Duarte MF Costa C Barbosa MA Pereira Dos Santos MC. Role of recombinant parvalbumin Gad c 1 in the diagnosis and prognosis of fish allergy. J Investig Allergol Clin Immunol. (2020) 30:340–5. 10.18176/jiaci.0437 31530508

[107] Borres MP Maruyama N Sato S Ebisawa M. Recent advances in component resolved diagnosis in food allergy. Allergol Int. (2016) 65:378–87. 10.1016/j.alit.2016.07.002 27543004

[108] Santos AF Alpan O Hoffmann HJ. Basophil activation test: mechanisms and considerations for use in clinical trials and clinical practice. Allergy. (2021) 76:2420–32. 10.22541/au.159493194.42634850 33475181

[109] Santos AF Shreffler WG. Road map for the clinical application of the basophil activation test in food allergy. Clin Exp Allergy. (2017) 47:1115–24. 10.1111/cea.12964 28618090 PMC5601249

[110] Imakiire R Fujisawa T Nagao M Tokuda R Hattori T Kainuma K. Basophil activation test based on CD203c expression in the diagnosis of fish allergy. Allergy Asthma Immunol Res. (2020) 12:641–52. 10.4168/aair.2020.12.4.641 32400130 PMC7224992

[111] Bahri R Custovic A Korosec P Tsoumani M Barron M Wu J. Mast cell activation test in the diagnosis of allergic disease and anaphylaxis. J Allergy Clin Immunol. (2018) 142:485–96.e16. 10.1016/j.jaci.2018.01.043 29518421 PMC6075471

[112] Jiménez-Saiz R Bruton K Koenig JFE Waserman S Jordana M. The IgE memory reservoir in food allergy. J Allergy Clin Immunol. (2018) 142:1441–3. 10.1016/j.jaci.2018.08.029 30201515

