# Corrigendum: Fish Allergy Around the World—Precise Diagnosis to Facilitate Patient Management

**DOI:** 10.3389/falgy.2021.797456

**Published:** 2021-11-08

**Authors:** Tanja Kalic, Christian Radauer, Andreas L. Lopata, Heimo Breiteneder, Christine Hafner

**Affiliations:** ^1^Department of Dermatology, University Hospital St. Poelten, Karl Landsteiner University of Health Sciences, St. Poelten, Austria; ^2^Center for Pathophysiology, Infectiology and Immunology, Institute of Pathophysiology and Allergy Research, Medical University of Vienna, Vienna, Austria; ^3^Molecular Allergy Research Laboratory, Australian Institute of Tropical Health and Medicine, James Cook University, Townsville, QLD, Australia; ^4^Tropical Futures Institute, James Cook University, Singapore, Singapore; ^5^Karl Landsteiner Institute for Dermatological Research, Karl Landsteiner Society, St. Poelten, Austria

**Keywords:** fish allergy, fish allergen diversity, fish extracts, molecular allergy diagnosis, patient management, cross-reactivity

There is an error in the Funding statement, funding source “the Austria Science Fund (FWF) grant P 30936-B30” was incorrectly written as “the Austria Science Fund (FWF) grant B 30936-B30” The corrected Funding statement appears below.

Funding for this work was provided by the Danube Allergy Research Cluster of Lower Austria (P-06), the Medical University of Vienna, the Austria Science Fund (FWF) grant P 30936-B30, and the NHMRC grant APP1086656.

The authors apologize for this error and state that this does not change the scientific conclusions of the article in any way. The original article has been updated.

## Publisher's Note

All claims expressed in this article are solely those of the authors and do not necessarily represent those of their affiliated organizations, or those of the publisher, the editors and the reviewers. Any product that may be evaluated in this article, or claim that may be made by its manufacturer, is not guaranteed or endorsed by the publisher.

